# Trend and forecast analysis of the changing disease burden of pancreatic cancer attributable to high fasting glucose in China, 1990–2021

**DOI:** 10.3389/fonc.2024.1471699

**Published:** 2024-10-18

**Authors:** Lichen Song, Ziyi Chen, Yongjie Li, Lirong Ran, Dongwei Liao, Yuanyuan Zhang, Guangming Wang

**Affiliations:** ^1^ School of Clinical Medicine, Dali University, Dali, Yunnan, China; ^2^ Medicine Department, School of Clinical Medicine, Dali University, Dali, Yunnan, China; ^3^ Center of Genetic Testing, The First Affiliated Hospital of Dali University, Dali, Yunnan, China

**Keywords:** pancreatic cancer, high fasting glucose, burden of disease, prediction, China

## Abstract

**Background:**

Pancreatic cancer (PC) is a malignant tumour with poor prognosis and high mortality, and high fasting plasma glucose (HFPG) is considered to be one of its important risk factors.

**Methods:**

PC disease burden data were obtained from the Global Burden of Disease Study 2021 (GBD 2021) database. Annual percent change (APC), average APC (AAPC), and 95% confidence interval (95% CI) were analysed using joinpoint linkpoint regression models to assess the trend of PC burden of disease between 1990 and 2021. An age-period-cohort model was used to estimate the independent effects of age, period, and cohort on PC burden, and data on PC mortality attributable to HFPG in China from 2022 to 2032 were analysed on the basis of a Bayesian age-period-cohort model projection.

**Results:**

The number of Pc deaths due to HFPG continue to rise in China from 1990 to 2021, with age-standardised mortality (ASMR) and age-standardised disability-adjusted life-year rates with increasing AAPC values of 1.12% (95% CI, 0.73–1.52) and 1.00% (95% CI, 0.63–1.37), respectively. Throughout the study, we found that the overall level of PC disease burden was significantly higher in men than that in women. In age-period-cohort analyses, the age effect of PC showed an increasing and then decreasing trend, the period effect showed an overall increasing trend during the study period, and the cohort effect showed an overall slow decreasing trend. In addition, the BAPC model predicted that ASMR is expected to decline significantly in both men and women from 2022 to 2032.

**Conclusions:**

It was found that PC attributable to HFPG was generally on the rise in China from 1990 to 2021 and has been on the decline in recent years, and projections suggest that the country’s future PC disease burden will continue to show a downward trend. Age and period of birth are the main factors affecting the disease burden, especially in men and older age groups. Early prevention, regular screening, and research into the pathogenesis of PC have, therefore, become particularly important.

## 1 Introduction

Pancreatic cancer (PC) is a malignant tumour with a poor prognosis and a very high mortality rate. According to the WHO Global Cancer Report 2020 (GLOBOCAN 2020), approximately 500,000 people worldwide are newly diagnosed with PC, with a similar number of deaths, and PC is the 12th most prevalent cancer in the world and the 7th leading cause of mortality (1). In recent years, the morbidity and mortality of PC in China are in the stage of increasing, accounting for the 7th place among malignant tumours for men and the 11th place for women, and the morbidity and mortality of PC gradually increase with the increase of age. PC has a relatively high degree of malignancy, and the condition progresses rapidly, but, due to the insidious onset of the disease and the atypical early symptoms, most of the patients have already belonged to the middle and late stages when they visit the doctor (2). PC has a very poor prognosis, with a 5-year relative survival rate of only 10% (3). PC projected to be third leading cause of cancer deaths by 2025 (4). Survival rates for patients with PC remain stagnant, despite the fact that survival rates for many tumours have now improved significantly (5); PC not only increases the economic burden on individuals and families but also becomes a major public health problem that threatens public health.

Epidemiological evidence supports type 2 diabetes mellitus as a risk factor for PC, and chronic hyperinsulinaemia and hyperglycaemia associated with type 2 diabetes mellitus have been suggested as potential mechanisms for this (6, 7). It has been shown that hyperglycaemia promotes the proliferation of PC cells and the increase of intracellular reactive oxygen species levels, which further contributes to the development of PC (8). A dose-response meta-analysis showed a 14% increase in PC risk for every 0.56 mmol/L (10 mg/dL) increase in fasting blood glucose level (9). GBD defines HFPG as fasting blood glucose greater than 86.4–97.2mg/dL. Data from the GBD study suggest that an estimated 419,300 cancer deaths and 8.6 million cancer disability-adjusted life years (DALYs) globally could be attributable to HFPG in 2019 and that, globally, the number of ASMR and ASDR attributable to HFPG for cancer increased by 27.8% and 24.5%, respectively (10).

The Global Burden of Disease 2021 (GBD 2021) database integrates estimates of the burden of disease for 369 diseases and injuries and 87 risk factors in 204 countries or regions around the world and provides a comprehensive assessment of the burden of disease across a range of indicators, including disease prevalence, morbidity, mortality, and DALYs (11). Current studies have investigated the disease burden of HFPG, leading to a variety of cancers. However, there are limited studies on the temporal and future trends of PC attributable to HFPG mortality in China. To explore the burden of PC attributable to HFPG in China, we comprehensively analysed the temporal trend of PC mortality in China from 1990 to 2021 based on the most recent data from GBD 2021 and further projected the future disease burden. We hope to provide a reference basis for preventing PC and making health decisions.

## 2 Materials and methods

### 2.1 Data source

The data in this study mainly come from The Global Health Data Exchange (GHDx), which has a high degree of data authenticity and credibility, using various national disease surveillance systems, cause of death registration and reporting systems, and injury detection systems as data sources. It is freely available worldwide through the official website of the Institute for Health Metrics and Evaluation at the University of Washington, USA (http://ghdx.healthdata.org/). Data on annual crude death rates, DALYs, and corresponding age-standardised rates were extracted from the GBD 2021 database, disaggregated by sex and age. Based on the theoretical framework of comparative risk assessment, counterfactual reasoning analyses are used to assume that the exposure levels of other risk factors remain unchanged, This is the population attributable fraction (PAF) (12). From the database, “China” was selected as the geographical location; “pancreatic cancer” was selected as the disease classification; “number, rate, and percent” and its standardised rate were selected as the metrics”; and “deaths and DALYs” were included as the risk indicators. “Percent” and its standardised rate were selected as the metrics; “Deaths and DALYs” were selected as the indicators for analysis”; “high fasting plasma glucose” was included as the risk factor: and “gender” was selected as the risk factor. Sex was selected as “male, female, and both,” and year was selected as “1990–2021.” Because the number of patients with PC under 25 years of age is low, the age groups for this study were “25–84 years,” with one age group for every 5 years, for a total of 12 age groups. To project PC mortality due to HFPG in China, we also obtained population estimates for 2017–2100 from GBD 2021 (https://ghdx.healthdata.org/record/ihme-data/global-population-forecasts-2017-2100).

### 2.2 Method

#### 2.2.1 Joinpoint regression analysis

Data were data collated and analysed using R studio, and line and histograms were plotted for data visualisation. Regression analysis of temporal trends was performed using Joinpoint Regression Program 4.7.0.0 software, which is a model based on the temporal characteristics of the disease to find statistically significant inflection points through the Monte Carlo substitution test (*P <* 0.05). The long-term temporal changes are divided into intervals, and, then, each specific interval is fitted and analysed to specifically characterise the epidemiology of a disease. Annual percent change (APC), average APC (AAPC), and 95% confidence interval (95% CI) were calculated, with APC > 0 indicating an increase in the interval from year to year and APC < 0 indicating a decrease in the interval from year to year; all tests were two-sided (13).

#### 2.2.2 Age-period-cohort models analysis

The APC model is based on the Poisson distribution (14). A commonly used tool in demographics and epidemiology to determine long-term trends in disease mortality. Since an individual’s birth cohort is determined using the time period of death and the individual's age at death (birth cohort = period - age). Taking every 5 years as an age group, the population included in this study was 25–84 years old and was divided into 12 groups. To satisfy the structural requirements of the model for age group spacing = period group spacing, the years 1990–2021 were divided into six groups using the same 5-year spacing, and the birth cohort was calculated by subtracting the age from the period to obtain a total of 17 cohorts. Because age, period, and cohort have a perfectly linear relationship, there is the problem of model non-identification. The intrinsic estimator was applied to estimate the effect coefficients to further calculate the relative risk (RR) of mortality, RR = EXP (effect coefficient) (15). In this study, Stata 17.0 was used to construct an APC model to describe the mortality rate of PC attributable to HFPG and its changes in China through effect coefficients, with positive coefficients indicating an increase in the mortality rate and with negative values indicating a decrease in the mortality rate. Excel 2019 software was used for data collation and GraphPad Prism 9 software was used for graphing.

#### 2.2.3 Mortality estimates by the Bayesian age-period-cohort model

R packages “BAPC” and “INLA (integrated nested Laplace approximation)” were used to implement BAPC, relying on the INLA algorithm to directly approximate the posterior marginal distribution (16, 17), forecasting data on PC attributable to HFPG in China, 2022–2032, with graphs using the ggplot2 package.

## 3 Results

### 3.1 Trends in PC deaths and DALYs attributable to HFPG in China, 1990–2021

**Figure 1:**
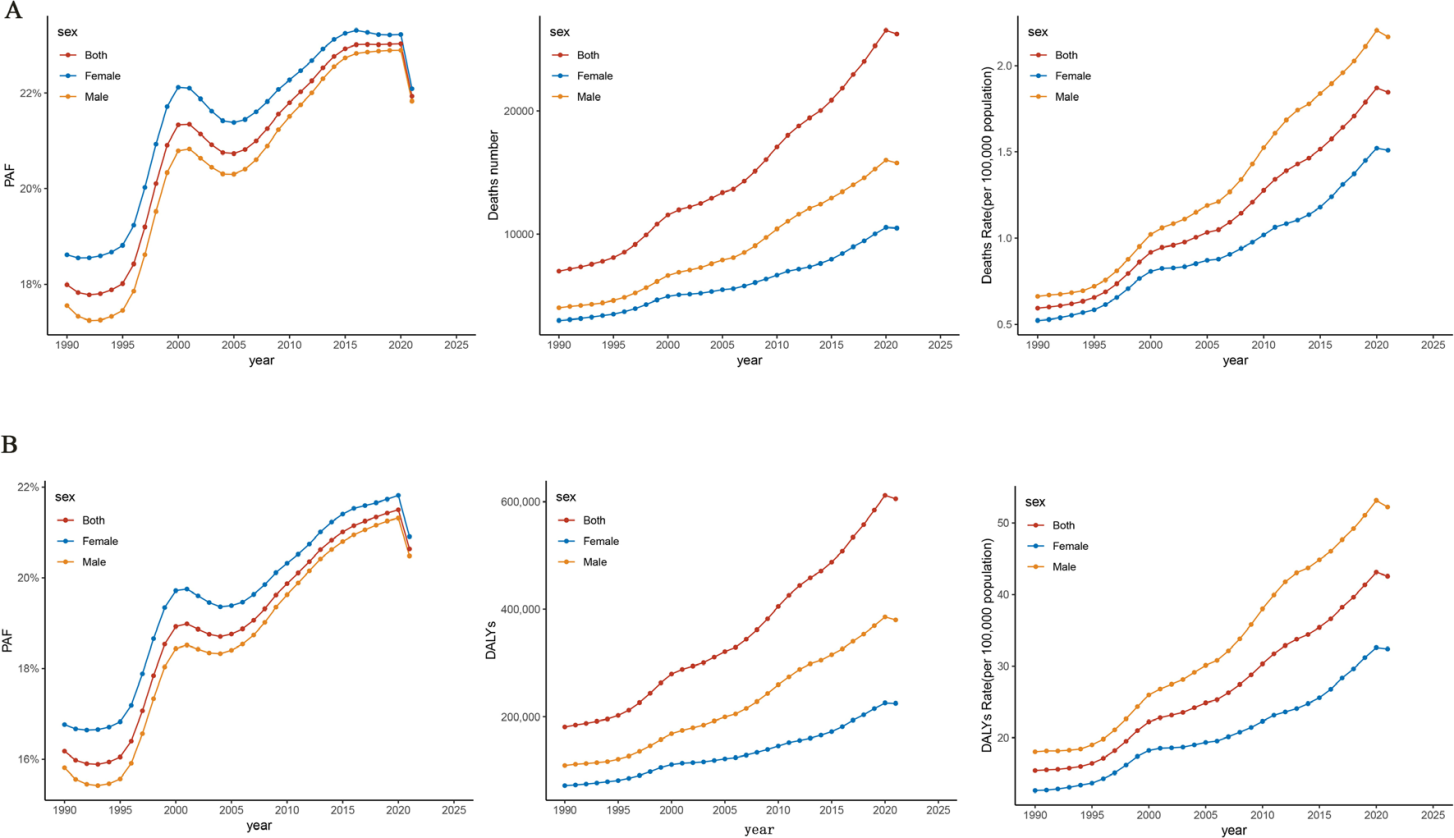
Trends in pancreatic cancer disease burden attributable to HFPG, China, 1990–2021. **(A)** Trends in pancreatic cancer deaths attributable to HFPG. **(B)** Trends in pancreatic cancer DALYs attributable to HFPG. HFPG, high fasting plasma glucose; PAF, population attributable fraction; DALYs, disability-adjusted life years.

The number of deaths and mortality rate of PC attributable to HFPG in China from 1990 to 2021 showed an overall increasing trend year by year, with the number of deaths increasing from 6,754 cases in 1990 to 26,256 cases in 2021 and with the mortality rate increasing from 0.59/100,000 in 1990 to 1.85/100,000 in 2021, respectively. Our results showed that the number of PC deaths and mortality rates attributed to HFPG increased with increasing years, and the trends were similar for men and women, but a decrease in the number of deaths and mortality rates could be observed in 2021 (**Figure 1A**). The rates of DALYs and DALYs of PC attributed to HFPG in China showed a rising trend from 1990 to 2021, with DALYs increasing from 181,128 in 1990 to 605,286 DALYs in 2019, and the rate of DALYs increased from 15.40/100,000 in 1990 to 42.54/100,000 in 2019 (**Figure 1B**).

### 3.2 Changes in time trends of joinpoint regression of ASMR and ASDR for PC attributable to HFPG in China, 1990–2021

**Figure 2:**
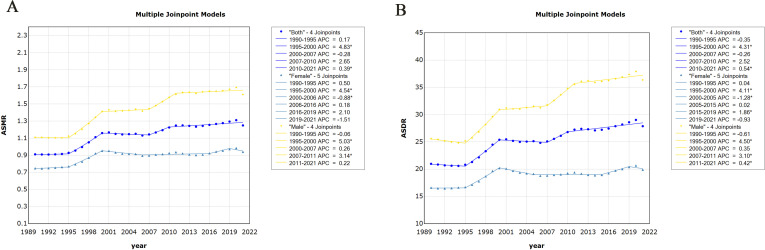
Changes in time trends of joinpoint regression of pancreatic cancer attributable to HFPG in China, 1990–2021. **(A)** Time trends in age-standardised mortality rates. **(B)** Time trends in age-standardised disability-adjusted life years. HFPG, high fasting plasma glucose; ASMR, age-standardised mortality rates; ASDR, age-standardised disability-adjusted life-year rates.

The joinpoint regression model plot of ASMR of PC attributable to HFPG in China from 1990 to 2021 contained four turning points divided into five segments: 1990–1995, APC = 0.17%; 1995–2000, APC = 4.83%; 2000–2007, APC = −0.28%; 2007–2010, APC = 2.65%; and 2010–2021, APC = 0.39%, all of which are increasing except for the 2000–2007 ASMR, which shows a downward trend (**Figure 2A**). Male mortality increased by an average of 1.32% per year (95% CI, 1.03–1.60; *P* < 0.05), and female mortality increased by an average of 0.79 per year (95% CI, 0.35–1.23, *P* < 0.05). Overall, the ASMR of PC attributable to HFPG increased at an average annual rate of 1.12% (AAPC = 1.12%; 95% CI, 0.73 to 1.52; *P* < 0.05) in China from 1990 to 2021 (**Table 1**). The joinpoint regression model plot of the ASDR for PC attributable to HFPG in China from 1990 to 2021 contained four turning points divided into five segments: 1990–1995, APC = 0.35%; 1995–2000, APC = 4.31%; 2000–2007, APC = −0.26%; 2007–2010, APC = 2.52%; and 2010–2021, APC = 0.54%, all of which showed an increasing trend except for the 2000–2007 ASDR, which showed a decreasing trend (**Figure 2B**). ASDR increased by an average of 1.22% per year (95% CI, 0.96–1.49; *P* < 0.05) for men and 0.63% per year (95% CI, 0.33–0.93; *P* < 0.05) for women. Overall, the ASDR for PC attributable to HFPG increased at an average annual rate of 1.00% (AAPC = 1.00%; 95% CI, 0.63 to 1.37; *P* < 0.05) in China from 1990 to 2021 (**Table 2**).

**Table 1:** Time trends in age-standardised mortality from pancreatic cancer attributable to high fasting glucose in China.

	Trends	Year	APC	95% CI	*P*	AAPC	95% CI	*P*
Female								
	1	1990–1995	0.50	(−0.24, 1.25)	0.17	0.79	(0.35, 1.23)	<0.05
	2	1995–2000	4.54	(3.46, 5.64)	<0.05			
	3	2000–2006	−0.88	(−1.62, −0.14)	<0.05			
	4	2006–2016	0.18	(−0.12, 0.47)	0.22			
	5	2016–2019	2.10	(−1.22, 5.54)	0.20			
	6	2019–2021	−1.51	(−4.78, 1.87)	0.35			
Male								
	1	1990–1995	−0.06	(−0.73, 0.61)	0.84	1.32	(1.03, 1.60)	<0.05
	2	1995–2000	5.03	(4.03, 6.03)	<0.05			
	3	2000–2007	0.26	(−0.25, 0.76)	0.30			
	4	2007–2011	3.14	(1.63, 4.67)	<0.05			
	5	2011–2021	0.22	(−0.02, 0.45)	0.07			
Both								
	1	1990–1995	0.17	(−0.58, 0.93)	0.64	1.12	(0.73, 1.52)	<0.05
	2	1995–2000	4.83	(3.70, 5.96)	<0.05			
	3	2000–2007	−0.28	(−0.85, 0.30)	0.32			
	4	2007–2010	2.65	(−0.72, 6.14)	0.12			
	5	2010–2021	0.39	(0.17, 0.62)	<0.05			

**Table 2:** Time trends in age-standardised disability-adjusted life years from pancreatic cancer attributable to high fasting glucose in China.

	Trends	Year	APC	95% CI	*P*	AAPC	95% CI	*P*
Female								
	1	1990–1995	0.04	(−0.53, 0.62)	0.15	0.63	(0.33, 0.93)	<0.05
	2	1995–2000	4.11	(3.26, 4.96)	10.50			
	3	2000–2005	−1.28	(−2.08, −0.47)	<0.05			
	4	2005–2015	0.02	(−0.21, 0.25)	0.14			
	5	2015–2019	1.86	(0.58, 3.16)	3.10			
	6	2019–2021	−0.93	(−3.48, 1.69)	<0.05			
Male								
	1	1990–1995	−0.61	(−1.23, 0.01)	0.05	1.22	(0.96, 1.49)	<0.05
	2	1995–2000	4.50	(3.58, 5.43)	<0.05			
	3	2000–2007	0.35	(−0.12, 0.81)	0.14			
	4	2007–2011	3.10	(1.71, 4.51)	<0.05			
	5	2011–2021	0.42	(0.20, 0.63)	<0.05			
Both								
	1	1990–1995	−0.35	(−1.07, 0.37)	0.32	1.00	(0.63, 1.37)	<0.05
	2	1995–2000	4.31	(3.24, 5.39)	<0.05			
	3	2000–2007	−0.26	(−0.8, 0.28)	0.33			
	4	2007–2010	2.52	(−0.66, 5.80)	0.11			
	5	2010–2021	0.54	(0.33, 0.76)	<0.05			

### 3.3 Trends in PC deaths and DALYs attributable to HFPG in different age groups

**Figure 3:**
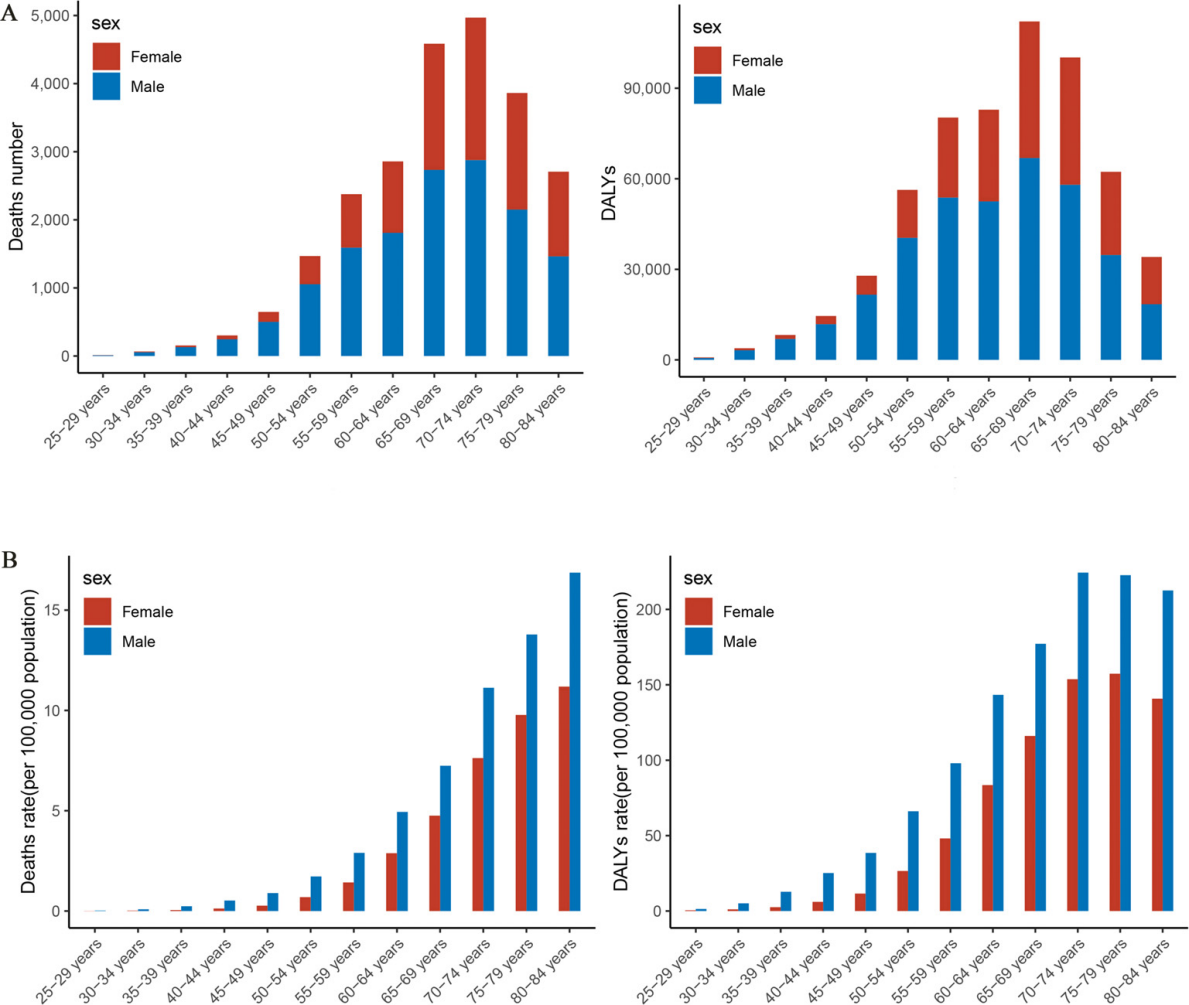
Trends in the burden of disease attributable to high fasting glucose in pancreatic cancer by age group and gender in China, 2021. **(A)** Trend changes in mortality, DALYs by age in 2021. **(B)** Trend changes in mortality, DALY rates by age in 2021. DALYs, disability-adjusted life years.

In 2021, the number of PC deaths attributable to HFPG among Chinese residents peaks in the 70–74 age group, whereas DALYs peak in the 65–69 age group, at 4,969 cases and 112,110 person-years, respectively (**Figure 3A**). Mortality rates continue to increase with age, especially after 50 years of age, peaking at 80–84 years of age. The rate of DALYs peaks at 75–79 years of age and then begins to decline (**Figure 3B**). Mortality and DALY rates for PC attributable to HFPG in 2021 followed the same trend with age.

### 3.4 Age-period-cohort effects

**Figure 4:**
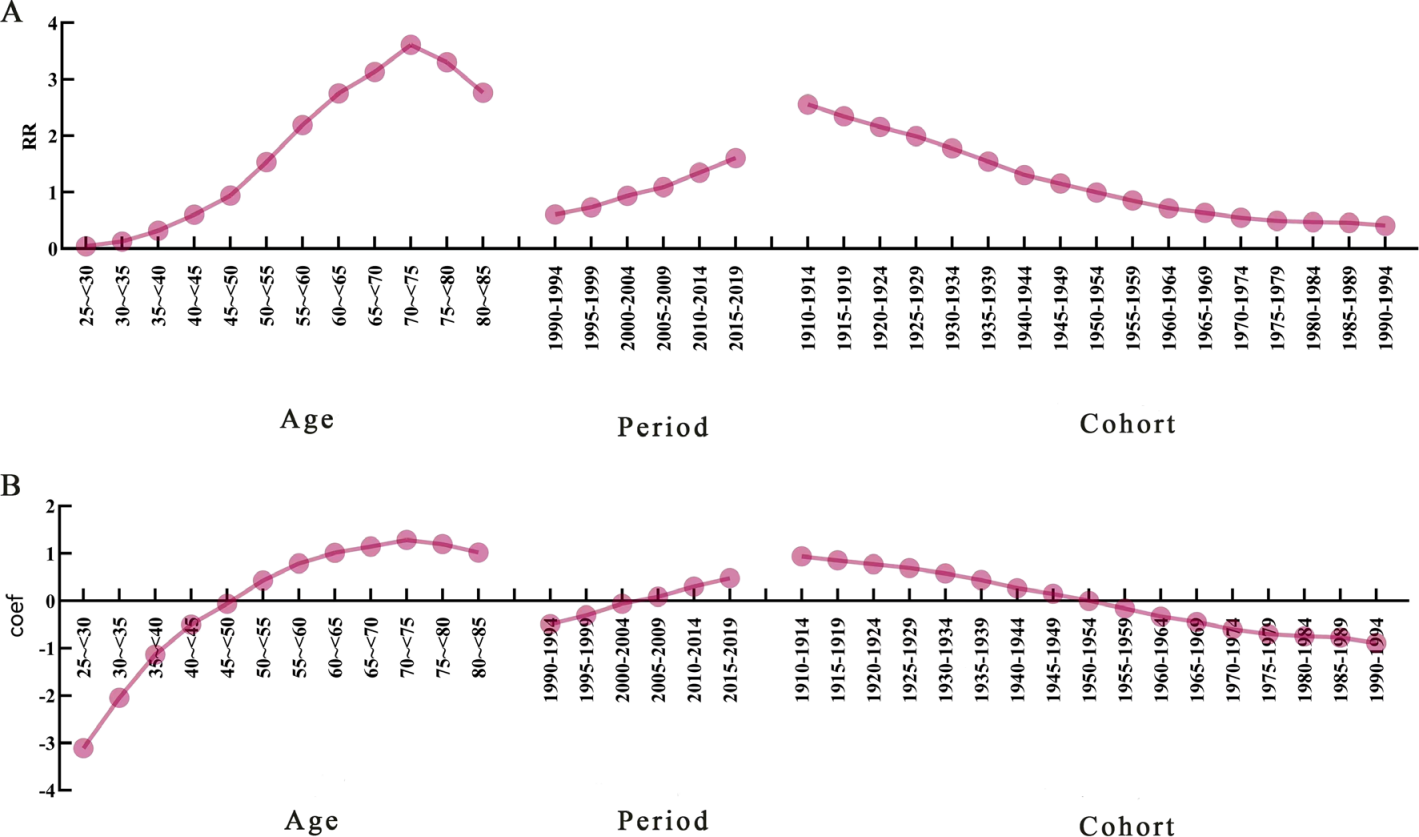
Age-period-cohort model analysis of pancreatic cancer mortality attributable to high fasting glucose. **(A)** Trends in the risk of death attributable to high fasting glucose in China caused by different effects. **(B)** Trends in mortality effect coefficients. RR, relative risk; Coef, coefficient of influence.

Age-period-cohort modelling was used to assess the effect of age, period, and cohort on deaths attributed to HFPG in patients with PC. The APC model decomposes mortality along three dimensions—age, period, and cohort—and analyses the effects of these three factors on mortality simultaneously. The age effect reveals different risks for various outcomes at different times of life, the period effect indicates population exposure at a defined point in time, and the cohort effect essentially represents differences in risk between birth cohorts. The effect coefficients and estimated RR for age, period, and cohort are shown in (**Figure 4**; **Table 3**).

**Table 3:** Results of age-period-cohort model analysis of pancreatic cancer mortality attributable to high fasting glucose.

Group	Coef (95% CI)	RR (95% CI)	SE	Z	*P*
Age					
age_25	−3.113 (−3.142, −3.085)	0.044 (0.043, 0.046)	0.014	−215.220	<0.001
age_30	−2.051 (−2.066, −2.035)	0.129 (0.127, 0.131)	0.008	−255.450	<0.001
age_35	−1.134 (−1.145, −1.123)	0.322 (0.318, 0.325)	0.006	−200.380	<0.001
age_40	−0.505 (−0.514, −0.497)	0.603 (0.598, 0.609)	0.005	−111.810	<0.001
age_45	−0.060 (−0.068, −0.053)	0.941 (0.935, 0.949)	0.004	−15.850	<0.001
age_50	0.428 (0.422, 0.434)	1.534 (1.524, 1.544)	0.003	134.480	<0.001
age_55	0.785 (0.780, 0.791)	2.193 (2.182, 2.205)	0.003	287.580	<0.001
age_60	1.013 (1.008, 1.017)	2.753 (2.740, 2.766)	0.002	423.270	<0.001
age_65	1.143 (1.138, 1.147)	3.135 (3.122, 3.149)	0.002	509.960	<0.001
age_70	1.284 (1.280, 1.288)	3.611 (3.595, 3.627)	0.002	570.120	<0.001
age_75	1.195 (1.190, 1.200)	3.303 (3.286, 3.319)	0.003	465.650	<0.001
age_80	1.016 (1.010, 1.023)	2.763 (2.745, 2.781)	0.003	307.880	<0.001
Period					
period_1990	−0.494 (−0.499, −0.490)	0.610 (0.607, 0.613)	0.002	−203.410	<0.001
period_1995	−0.305 (−0.309, −0.301)	0.737 (0.735, 0.740)	0.002	−149.870	<0.001
period_2000	−0.062 (−0.065, −0.058)	0.940 (0.937, 0.943)	0.002	−36.140	<0.001
period_2005	0.087 (0.083, 0.090)	1.090 (1.087, 1.094)	0.002	53.640	<0.001
period_2010	0.300 (0.296, 0.303)	1.349 (1.345, 1.354)	0.002	181.160	<0.001
period_2015	0.474 (0.471, 0.478)	1.607 (1.601, 1.612)	0.002	259.230	<0.001
Cohort					
cohort_1910	0.939 (0.919, 0.959)	2.557 (2.506, 2.609)	0.010	91.130	<0.001
cohort_1915	0.852 (0.840, 0.864)	2.344 (2.316, 2.372)	0.006	138.320	<0.001
cohort_1920	0.771 (0.762, 0.780)	2.161 (2.142, 2.181)	0.005	169.210	<0.001
cohort_1925	0.690 (0.683, 0.698)	1.994 (1.979, 2.009)	0.004	183.830	<0.001
cohort_1930	0.575 (0.569, 0.582)	1.778 (1.766, 1.789)	0.003	173.500	<0.001
cohort_1935	0.435 (0.429, 0.441)	1.545 (1.536, 1.555)	0.003	141.010	<0.001
cohort_1940	0.267 (0.260, 0.273)	1.306 (1.297, 1.314)	0.003	82.980	<0.001
cohort_1945	0.144 (0.137, 0.150)	1.154 (1.147, 1.162)	0.003	42.200	<0.001
cohort_1950	−0.001 (−0.008, 0.006)	0.999 (0.992, 1.006)	0.004	−0.190	0.846
cohort_1955	−0.161 (−0.169, −0.153)	0.852 (0.845, 0.858)	0.004	−39.670	<0.001
cohort_1960	−0.333 (−0.342, −0.324)	0.717 (0.710, 0.723)	0.005	−72.420	<0.001
cohort_1965	−0.450 (−0.459, −0.440)	0.638 (0.632, 0.644)	0.005	−89.910	<0.001
cohort_1970	−0.603 (−0.614, −0.592)	0.547 (0.541, 0.553)	0.006	−103.470	<0.001
cohort_1975	−0.706 (−0.720, −0.692)	0.494 (0.487, 0.501)	0.007	−97.280	<0.001
cohort_1980	−0.751 (−0.770, −0.732)	0.472 (0.463, 0.481)	0.010	−76.690	<0.001
cohort_1985	−0.775 (−0.803, −0.748)	0.461 (0.448, 0.474)	0.014	−55.260	<0.001
cohort_1990	−0.893 (−0.958, −0.827)	0.410 (0.384, 0.437)	0.033	−26.670	<0.001
_cons	−8.800 (−8.804, −8.795)	0.000 (0.000, 0.000)	0.002	−3605.520	<0.001

#### 3.4.1 Effect of age

After controlling for period and cohort effects, the age effect of the risk of death from PC attributable to HFPG in our population aged 25–84 years during the observation period first increased and then decreased with age. The lowest risk of death was found in the age group of 25 to <30 years, RR = 0.044 (0.043, 0.046), with an effect coefficient for age of −3.113. The highest value was reached in the age group of 70 to <75 years, RR = 3.611 (3.595, 3.627), with an age effect coefficient of 1.284, which increased the effect coefficient by 4.397 compared to the age group with the lowest risk of death, an almost 81-fold increase in the risk of death from PC, and then decreased rapidly to the age group of 80 to <84 years.

#### 3.4.2 Effect of period

The trend of the period effect coefficient of the risk of death attributable to HFPG in PC in China during the observation period showed an increasing trend in the risk of death. Years 1990–1994 had the lowest risk of death in the period group, RR = 0.610 (0.607, 0.613), with a period effect coefficient of −0.494, and years 2015–2019 had the highest risk of death in the period group, RR = 1.607 (1.601, 1.612), a period effect coefficient of 0.474, a period effect coefficient increase of 0.968, and a 2.63-fold increase in the risk of death.

#### 3.4.3 Effect of period cohort

With the development of the birth cohort during the observation period, our PC attributable to HFPG mortality risk of the cohort effect coefficient of the trend of change in the risk of death in the overall slowly decreasing trend. Years 1910–1994 birth cohort PC mortality risk had the largest RR = 2.557 (2.506, 2.609), with cohort effect coefficient of 0.939, in 1990–1994. The birth cohort had the smallest risk of death RR = 0.410 (0.384, 0.437), with a coefficient of coefficient of effect of −0.893, and the risk of death from PC in the 1910–1994 birth cohort was 6.25 times greater than that in the 1990–1994.

### 3.5 Trends in PC deaths attributable to HFPG in China, 2022–2032

**Figure 5:**
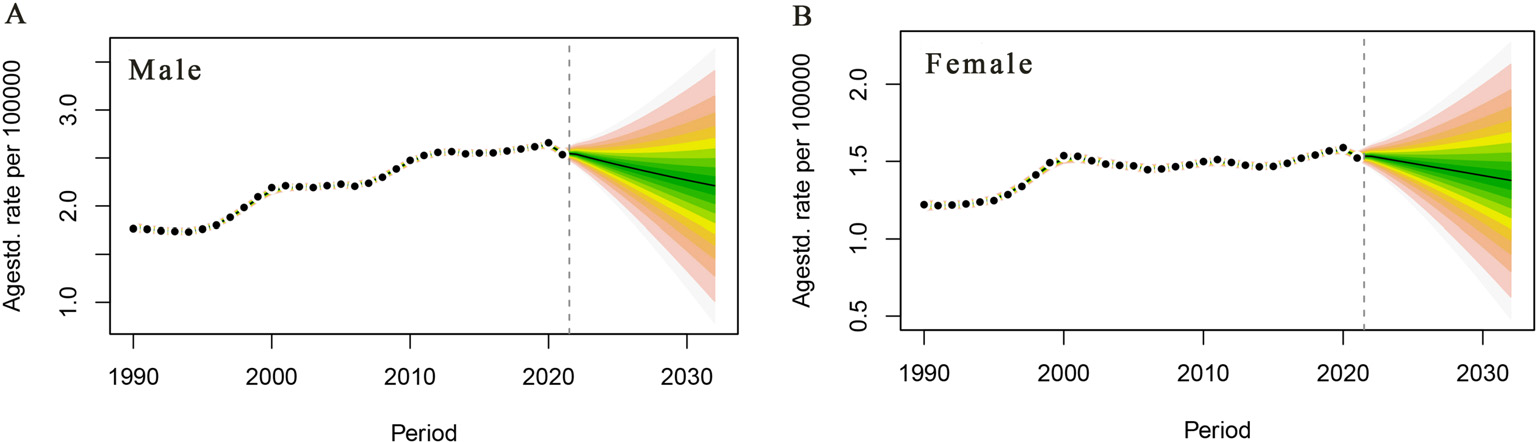
Trends in pancreatic cancer deaths attributable to high fasting glucose in China, 2022–2032. **(A)** Male age-standardised death rate. **(B)** Female age-standardised death rate.

PC mortality attributable to HFPG in the male population is projected to continue to decline over the next 10 years, with a male PC mortality rate of 2.21/105 in 2032, a decrease of 13.33% compared with that of 2.55/105 in 2021 (**Figure 5A**). PC mortality attributable to HFPG is projected to decrease steadily in the female population of China, with a projected female PC mortality rate of 1.38/105 in 2032, a decrease of 9.80% compared with that of 1.53/105 in 2021(**Figure 5B**).

## 4 Discussion

PC is a malignant tumour with a very high lethality rate. Due to its insidious onset and rapid progression, early detection is more difficult, and about 80% of patients are already in the progressive or advanced stage when they are detected, thus losing the best time for treatment (18). While the global population has increased by 44.67% over the past 30 years, the number of cancer deaths due to HFPG has increased by 179.37%, which is approximately four times the rate of population growth, and this is a serious global public health problem (19). In China, the incidence and mortality rates of PC are low overall, but they are still at an increasing stage, seriously affecting the health of the population.

Possible reasons for the increase in the disease burden of PC deaths are as follows: rapid socio-economic development, population ageing, and increased levels of PC risk factors; continuous improvement of medical technology, and increased detection rates of PC; increased health awareness of the population; and improved quality of tumour registries. Less research has been invested than in other malignant tumours, leading to increased disease burden. It is also related to the rapid socio-economic development and ageing of the population, which increases the level of risk factors associated with PC; the increasing detection rate of PC due to the continuous improvement of medical technology; the increasing health awareness of the population; the improvement of the quality of tumour registries; and the limited development of diagnostic and treatment techniques for PC due to the low investment in research compared with that for other malignant tumours (20, 21). Risk factors associated with the development of PC are varied and complex. Studies have shown that PC morbidity and mortality are associated with poor lifestyle habits, such as smoking, alcohol consumption, obesity, and dietary imbalances, all of which increase the risk of PC. In addition, numerous studies have found that the development of PC is also associated with age, gender, blood type, family history, genetic history, and race. Diabetes, chronic pancreatitis, infections, and intestinal dysbiosis also contribute to the increased risk of PC. Most malignant tumours show a clear positive correlation with age; the older the age, the higher the incidence of malignant tumours; PC is no exception, with the majority of patients occurring between 70 and 90 years old (22). The incidence and mortality rates of PC in the male population are 1.46 times higher than those in the female population, which may be related to the fact that the male population is more likely to be exposed to risk factors such as smoking and alcohol consumption (1). Studies have shown that the risk of PC development is related to ABO blood type, and a study from Wolpin et al. (23) showed that patients with blood types A, AB, and B had a significantly higher risk of PC compared to those with blood type O. Genetic factors play an integral role in the development of malignant tumours, and epidemiological studies have confirmed that (23) PC is characterised by familial aggregation, as evidenced by the diagnosis of two or more first-degree relatives in the family and that approximately 10% of PC have a familial basis. Smoking is a risk factor for many malignant tumours, and PC is no exception, as Bosetti et al. (24) concluded in their study that current smokers have at least a 100% increased risk of PC compared to non-smokers and that the risk increases with the number of cigarettes smoked and the duration of the cigarettes smoked. There is still disagreement as to whether ethanol intake increases the risk of PC, with some studies showing no association between ethanol intake and PC risk (25), and the results from a pooled cohort analysis that included 2,187 cases of PC showed a significant 22% increase in the risk of PC in those who consumed ≥30 g of alcohol per day, compared with those in non-drinkers (26). Body mass index >25.0 kg/m^2^ was found to increase the risk of PC (27). Overseas studies have reported that dietary factors influence the risk of PC, and a high intake of antioxidant-rich vegetables and fruits is associated with a 38% and 29% reduction in the risk of PC, respectively (28). Consumption of red meat (especially cooked at high temperatures), processed meats, fried foods, and other foods that contain nitrosamines may increase the risk of PC, which may be associated with carcinogens in meat and nitrites or N-nitroso compounds used to preserve processed meats (29). Studies have shown that prolonged diabetic disease increases the risk of PC, and patients with type 1 or type 2 diabetes have a risk of PC that is 1.8 to 2 times higher than that of normal individuals (7, 30), and a retrospective analysis by Sharma et al. (31) found that patients with PC develop new-onset fasting blood glucose elevations on average 30 to 36 months prior to diagnosis, which may indicate that new-onset diabetes is an early PC, which led to the exploration of the feasibility of using HbA1c as a marker for early detection of PC. Studies have shown that patients with chronic pancreatitis have a significant 13-fold increased risk of developing PC compared to normal individuals, with approximately 5% of these patients eventually developing PC (32). A study by Memba et al. (33) showed that a decrease in the number of streptococci in the digestive tract and an increase in the number of Porphyromonas gingivalis increased the risk of PC. It has also been shown (34) that *Helicobacter pylori* or *hepatitis C* infection is a risk factor for PC.

By gender, there were significant gender differences in the trend of PC deaths. The study found that the number of deaths, mortality rates, DALYs, and DALYs of PC attributable to HFPG were overall higher in Chinese men than those in women from 1990 to 2021. In a retrospective cohort study in China, the likelihood of PC in men and women with type 2 diabetes mellitus was 2.97 and 2.68 times higher than that in the general population, with men being significantly more likely than women (35). It has also been shown that slightly more men than women are diagnosed with type 2 diabetes mellitus and fasting hyperglycaemia, which may be related to the higher levels of exposure to risk factors in male populations, such as obesity, smoking, excessive alcohol intake, and sedentary behaviour (36). Possibly due to differences in risk factor exposure and hormones between genders, Chinese men have a higher smoking rate than women, so smoking is likely to be an important cause of the higher incidence in men than in women (37). Secondly, although there are few studies on the cellular mechanisms underlying the differences in the incidence of PC between men and women, female hormones have been shown to play a protective role against PC in a study of diet and cancer in Malmö and also after receiving hormone replacement therapy (38), and increasing the number of births may also reduce the incidence of PC (39).

By age, both the mortality rate and DALY rate of PC attributable to HFPG in China in 2021 showed an increasing trend with age. The burden of disease attributable to PC varies among different age groups, and this study showed that age-specific mortality rates for PC increased continuously with age, especially after 50 years of age, with a rapid increase, peaking at 80–84 years of age. The first country possibly related to the ageing population is China, which now has the largest elderly population in the world. In 2019, the population aged 60 years and, over the country, was 254 million. By 2040, this is expected to increase to 402 million or about 28% of the population (40). It may be related to the fact that PC is a disease of ageing and may be associated with a decline in autoimmunity with age and an increased risk of developing PC. Secondly, studies have shown that older patients are often less receptive to PC treatment, with comorbidities, malnutrition, impaired physical and cognitive function, and limited social support leading to a worse prognosis (41). Therefore, precision prevention and treatment of PC should be personalised by formulating improvement measures for specific maladaptive behaviours of specific populations. For example, in the context of population ageing, geriatric health management should be strengthened, emphasis should be placed on the rehabilitation of chronic diseases and the prevention of serious complications, the severity of disease in elderly patients should be reduced, and the quality of life of elderly patients with PC should be improved. In addition, men and middle-aged and elderly people should be taken as the key targets of PC surveillance, and the allocation of health resources should be appropriately favoured.

In this paper, we used the APC model to analyse the independent effects of age, period, and birth cohort on the change in PC mortality attributable to HFPG in China from 1990 to 2021, where the burden of PC in the population is high and shows a gradual increase. Age effect response to physiological changes induced by ageing and cumulative exposure to risk factors on mortality from PC (42). Age effect plays an important role in the mortality of PC, and, during the observation period, the age effect of the mortality risk of 25- to 84-year-old people in China firstly increases and then slowly decreases with the increase of age and reaches the highest value in the age group of 70~<75 years old, and the majority of patients with PC are concentrated in the age group of 50~85 years old, which indicates that middle-aged and old people have higher possibilities of suffering from PC compared with young people, which may be due to the decrease of autoimmunity and the increase of risk of suffering from PC with the increase of age. This indicates that middle-aged and old people are more likely to develop PC than young people, which may be due to the decrease of autoimmunity with age and the increased risk of PC (43). This suggests a focus on early screening, prevention, and control strategies for the elderly population. Period effects are usually caused by complex historical events and environmental factors (44). The period effect coefficient and RR value of PC mortality attributable to HFPG in China during the observation period of the current study showed a continuous increasing trend, with the period effect coefficient increasing from −0.494 to 0.968 and with the risk of death increasing by 2.63 times. Since the 21st century, with the rapid development of medical diagnostic and treatment technology in China, the mortality rate of PC has been increasing, so there is an urgent need for early diagnosis and good therapeutic techniques to alleviate the burden of PC, but the progress in the past few decades is still slow (45). Cohort effects describe temporal differences in birth rates between groups born in the same time period, reflecting changes in lifestyle and exposure to risk factors over time (46, 47). After controlling for age and period factors, the results of the APC analysis show that the cohort effect coefficients are highest for the 1910–914 cohort and lowest for the 1990–1994 cohort, which implies that the younger the population, the smaller the effect of birth cohort on PC mortality. The possible reason for this is that the later birth cohort received better health education and, therefore, had a greater awareness of health and disease prevention compared to the earlier birth cohort (48).

In summary, the mortality and DALY rates PC among Chinese residents showed an overall increasing trend from 1990 to 2021, and attention should be paid to men and the elderly population. It is worth noting that the results of this projection show that the mortality rate of PC in women will be 1.38/105 in 2032, a decrease of 9.80% compared with that in 2021 (1.53/105), and the mortality rate of PC attributed to HFPG in male residents is predicted to continue to decrease in the next 10 years, and the mortality rate of PC in men will be 2.21/105 in 2032, a decrease of 13% compared with that in 2021 (2.55/105) by 13.33%. The analysis of the data in this paper shows that the incidence and mortality rates of PC are generally on the rise, but the number of deaths, mortality rates, DALYs, DALY rates, and PAF will have decreased significantly in 2021, which is correlated with the conclusion that the burden of PC in China is expected to continue to decline in the future, indicating that the burden of PC in China has changed significantly. This result may be due to the fact that, in the future, with the development of science and technology and social economy, China’s medical level will continue to improve, medical security will be gradually perfected, PC treatment modalities will be enriched and improved, the health awareness of the population will be raised, and the diagnosis and treatment capacity of PC in China has made obvious progress, thus making it possible to predict that the burden of PC disease will continue to decline in China in the future. However, due to the inadequacy of diagnosis and treatment in China in the early years, coupled with the failure of new therapeutic means such as immunological and targeted drugs to significantly improve the prognosis of PC, this may be one of the reasons for the continued increase in the mortality rate of PC in China today. At present, our country is implementing risk factor control actions to reduce the risk of cancer. In addition, there is a need to strengthen the dissemination of knowledge about PC prevention and treatment and the implementation of early diagnosis and treatment programmes in all areas, so as to achieve the goal of awareness of PC prevention and treatment and early diagnosis as soon as possible (49).

### 4.1 Limitations

There are some limitations of this study. Firstly, the data in this study were estimated by GBD 2021 based on relevant tumour data in China, which is not first-hand information, so it is difficult to avoid the possibility of distortion of the estimation results. Secondly, this study was a descriptive analysis of the overall PC attributable to HFPG in China and did not specifically analyse the differences between provinces, regions, and urban/rural areas in China. Lastly, the burden of disease of PC attributable to HFPG in China was analysed on the basis of the assumption that the risk factor HFPG has an independent effect. Finally, the burden of disease PC attributable to HFPG in China was analysed on the basis of the assumption that the risk factor HFPG has an independent effect, but HFPG usually coexists and interacts with other factors.

## 5 Conclusions

In conclusion, the situation of PC deaths and DALYs attributed to HFPG in our country should not be ignored. Early screening of high-risk groups for PC attributable to HFPG should focus on men and middle-aged and older populations. Early prevention, regular screening, and pathogenesis of PC have become particularly important due to the lack of specific treatment for PC. In conclusion, health policymakers should develop comprehensive strategies and measures to reduce the burden of PC caused by HFPG that are more appropriate to the country’s situation in order to prolong the life expectancy of the population.

## Data Availability

The original contributions presented in the study are included in the article/supplementary material. Further inquiries can be directed to the corresponding authors.
